# Transient acute kidney injury after chimeric antigen receptor T-cell therapy in patients with hematological malignancies

**DOI:** 10.1093/ckj/sfae027

**Published:** 2024-02-20

**Authors:** Juan León-Román, Gloria Iacoboni, Sheila Bermejo, Cecilia Carpio, Mónica Bolufer, Clara García-Carro, Mario Sánchez-Salinas, Carla Alonso-Martínez, Oriol Bestard, Pere Barba, María José Soler

**Affiliations:** Nephrology Department, Vall d'Hebron University Hospital, Vall d'Hebron Institute of Research, CSUR National Unit of Expertise for Complex Glomerular Diseases of Spain, Barcelona, Spain; Department of Hematology, Vall d'Hebron University Hospital, Experimental Hematology, Vall d'Hebron Institute of Oncology (VHIO), Vall d'Hebron Barcelona Hospital Campus, Passeig Vall d'Hebron, Barcelona, Spain; Department of Medicine, Universitat Autònoma de Barcelona, Bellaterra, Spain; N ephrology Department, Vall d'Hebron University Hospital, Vall d'Hebron Institute of Research, CSUR National Unit of Expertise for Complex Glomerular Diseases of Spain, Barcelona, Spain; Department of Hematology, Vall d'Hebron University Hospital, Experimental Hematology, Vall d'Hebron Institute of Oncology (VHIO), Vall d'Hebron Barcelona Hospital Campus, Passeig Vall d'Hebron, Barcelona, Spain; N ephrology Department, Vall d'Hebron University Hospital, Vall d'Hebron Institute of Research, CSUR National Unit of Expertise for Complex Glomerular Diseases of Spain, Barcelona, Spain; Nephrology Department, San Carlos Clinical University Hospital, Madrid, Spain; Department of Hematology, Vall d'Hebron University Hospital, Experimental Hematology, Vall d'Hebron Institute of Oncology (VHIO), Vall d'Hebron Barcelona Hospital Campus, Passeig Vall d'Hebron, Barcelona, Spain; Pharmacy Department, Vall d´Hebron Hospital Universitari, Vall d´Hebron Barcelona Hospital Campus, Barcelona, Spain; N ephrology Department, Vall d'Hebron University Hospital, Vall d'Hebron Institute of Research, CSUR National Unit of Expertise for Complex Glomerular Diseases of Spain, Barcelona, Spain; Department of Hematology, Vall d'Hebron University Hospital, Experimental Hematology, Vall d'Hebron Institute of Oncology (VHIO), Vall d'Hebron Barcelona Hospital Campus, Passeig Vall d'Hebron, Barcelona, Spain; N ephrology Department, Vall d'Hebron University Hospital, Vall d'Hebron Institute of Research, CSUR National Unit of Expertise for Complex Glomerular Diseases of Spain, Barcelona, Spain

**Keywords:** acute kidney injury, CAR-T therapy, onconephrology

## Abstract

**Background:**

Acute kidney injury (AKI) occurs in 30% of patients infused with chimeric antigen receptor (CAR) T-cells. The purpose of this study was to identify risk factors and long-term outcomes after AKI in patients who received CAR T-cell therapy.

**Methods:**

Medical records of 115 adult patients with R/R hematological malignancies treated with CD19-targeted CAR T-cells at Vall d'Hebron University Hospital between July 2018 and May 2021. Baseline demographic data including age, gender, ethnicity, body mass index (BMI), and co-morbidities, as well as the type of hematological neoplasia and prior lines of therapy were collected. Laboratory parameters including serum creatinine and whole blood hemoglobin were retrospectively reviewed and values were gathered for days +1, +7, +14, +21, and +28 post-infusion.

**Results:**

A total of 24/115 (21%) patients developed AKI related to CAR T-cell therapy; 6/24 with AKI over chronic kidney disease (CKD). Two patients had AKI in the context of lymphodepleting (LD) chemotherapy and the other 22 after CAR T-cell infusion, starting at day+1 in 3 patients, day+7 in 13 patients, day +14 in 1 patient, day+21 in 2 patients, and day+28 in 3 patients. Renal function was recovered in 19/24 (79%) patients within the first month after infusion. Male gender, CKD, cytokine release syndrome (CRS), and immune effector cell-associated neurotoxicity syndrome (ICANS) were associated with AKI. Male gender, CKD, ICANS grade ≥3 and CRS grade ≥2 were identified as independent risk factors for AKI on multivariable analysis. In terms of the most frequent CAR T-cell related complications, CRS was observed in 95 (82%) patients and ICANS in 33 (29%) patients. Steroids were required in 34 (30%) patients and tocilizumab in 37 (32%) patients. Six (5%) patients were admitted to the intensive care unit (1 for septic shock, 4 for CRS grade ≥2 associated to ICANS grade ≥2, and 1 for CRS grade ≥3). A total of 5 (4.4%) patients died in the first 30 days after CAR T-cell infusion for reasons other than disease progression, including 4 cases of infectious complications and 1 of heart failure.

**Conclusion:**

Our results suggest that AKI is a frequent but mild adverse event, with fast recovery in most patients.

## Graphical Abstract

**Graphical Abstract:**
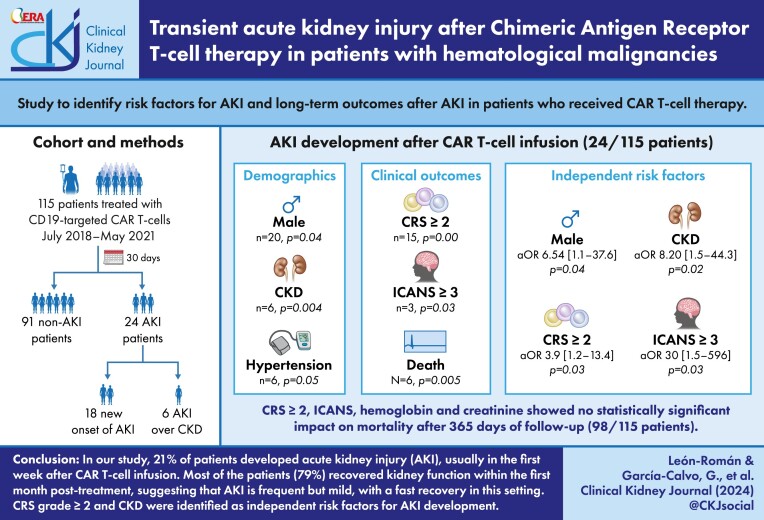

KEY LEARNING POINTS **What was known:**

- CAR T-cell therapy has increased survival of patients with relapsed/refractory hematological malignancies.
- Acute kidney injury (AKI) occurs in 20–30% of patients receiving CAR T-cell therapy.
- Risk factors for acute kidney injury development in the setting of CAR T-cell therapy are not completely established.

**This study adds:**

- Clinical characteristics of AKI in one of the largest series of hematological cancer patients treated with CAR T-cell therapy.
- We demonstrated that AKI is mainly a transient complication in patients treated with CAR T-cell therapy.
- In our cohort, males, CKD, ICANS, and CRS were identified as risk factors for AKI after CAR T-cell therapy.

**Potential impact:**

- Preventing and diagnosing AKI in patients treated with CAR T-cells.
- Strict monitoring of renal function in patients at risk, mainly male patients, previous history of CKD, and those developing CRS and ICANS after infusion.
- Early Nephrology referral in patients treated with CAR T-cells who develop AKI without a fast renal function recovery.

## INTRODUCTION

Chimeric antigen receptors (CARs) are engineered synthetic proteins that redirect the specificity of T cells [1, 2]. The structure of commercially available CARs includes extracellular immunoglobulin-derived heavy and light chains to recognize specific antigens, and intracellular activating and costimulatory domains to lead signal activation, cytokine release, T-cell proliferation, and immune cell response against tumor cells. [1, 3–5]

CAR T-cell therapy has been a revolutionary treatment for relapsed/refractory (R/R) hematological malignancies including adult and pediatric B-cell acute lymphoblastic leukemia, diffuse large B-cell lymphoma, follicular lymphoma and multiple myeloma [6–21]. Furthermore, there are promising results in selected solid tumors and autoimmune diseases [22, 23]. Response rates and long-term survival are variable across diseases but they outweigh the current available therapies in these indications.

CAR T-cell therapy has a well-known acute and long-term toxicity profile [24, 25]. The most frequent acute adverse event is cytokine release syndrome (CRS), occurring usually within the first week after CAR T-cell infusion [7–20, 22–30]. CRS manifests as a rapid immune reaction driven by the massive release of cytokines, including IFN-gamma and IL-6 [29–31]. The clinical presentation includes fever, hypotension, and/or hypoxia. Organ dysfunction can sometimes occur in this setting but does not impact CRS grading [29–32]. Immune effector cell-associated neurotoxicity syndrome (ICANS) is another frequent acute side effect which usually starts around the second week post-infusion, after CRS onset in the great majority of patients. Acute kidney injury (AKI) develops in 20–30% of infused patients, mainly associated with CRS, electrolyte disorders and tumor lysis syndrome (TLS) [33–35]. Regarding late complications, after the first month post-infusion, the most common are hypogammaglobulinemia, cytopenia, and infections [24–28, 29].

There is limited literature regarding AKI after CAR-T cell therapy. Kanduri *et al.* described in a systematic review of 22 cohort studies, a population of 3376 pediatric and adult patients that included the incidence of AKI and clinical complications after CAR T-cell infusion. The pooled estimated incidence of AKI was 18.6%, and AKI was presented in 17% in the subgroup of adults after the treatment. The estimated CRS incidence in all included studies was 75.4% [36]. The purpose of this study is to determine the demographics, laboratory results and clinical evolution of patients who received CAR T-cell therapy and developed AKI, as well as identifying potential risk factors.

## MATERIALS AND METHODS

### Data collection and analysis

**Figure 1:**
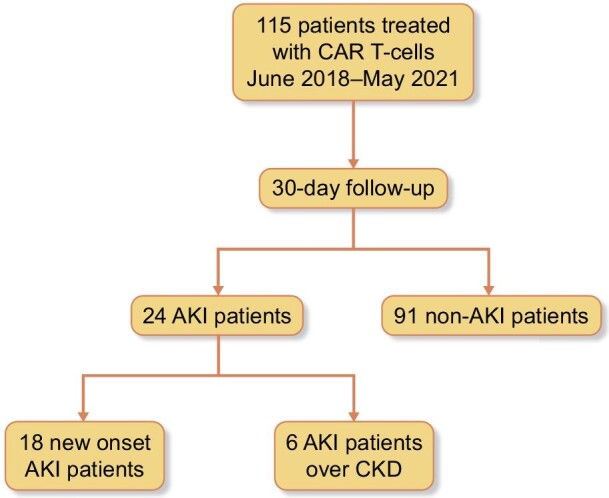
Flow diagram for study selection.

We conducted a retrospective review of the medical records of 115 adult patients with R/R hematological malignancies treated with CD19-targeted CAR T-cells at Vall d'Hebron University Hospital between July 2018 and May 2021 (Fig. 1). Data collection was performed with the approval of the Ethics Committee of the Vall d'Hebron University Hospital EOM(AG)043/2021(5853).

Baseline demographic data including age, gender, ethnicity, body mass index (BMI), and co-morbidities, as well as the type of hematological neoplasia and prior lines of therapy were collected. Laboratory parameters including serum creatinine and whole blood hemoglobin were retrospectively reviewed and values were gathered for days +1, +7, +14, +21, and +28 post-infusion.

### Definition and endpoints

AKI was defined according to the Kidney Disease Improving Global Outcomes (KDIGO) criteria: grade 1, increase in serum creatinine (SCr) 1.5 to <2-fold of baseline; grade 2, increase in SCr 2- to <3-fold of baseline; grade 3, increase in SCr ≥3-fold of baseline, OR requiring renal replacement therapy, OR SCr ≥4.0 mg/dL [37]. Chronic kidney disease (CKD) was defined according KDIGO as kidney damage or glomerular filtration rate (GFR) <60 ml/min/1.73 m^2^ with CKD-EPI formula for 3 months or more, irrespective of cause [38]. Recovery of renal function was established following the consensus report of the Acute Disease Quality Initiative (ADQI) [39]. Severe electrolyte disorders were defined according to the Common Terminology Criteria for Adverse Events (CTCAE) [40].

CRS and ICANS were graded according to the American Society of Transplantation and Cellular Therapy (ASTCT) criteria. Other treatment-related adverse events such as infections intensive care unit (ICU) admission and death were also recorded [41, 42].

### Lymphodepleting chemotherapy

All patients received LD chemotherapy on the week before CAR T-cell infusion. LD chemotherapy included cyclophosphamide and fludarabine for 3–4 consecutive days, according to the label recommendations for each disease.

Fludarabine required dose adjustment in case of a decreased creatinine clearance (CrCl), according to institutional guidelines. In brief: (i) if CrCl ≥70 mL/min/1.73 m^2^, no dose reduction was applied; (ii) if CrCl was 45–70 mL/min/1.73 m^2^, each daily dose was reduced by 25%; and (iii) if CrCl was 30–45 mL/min/1.73 m^2^, each daily dose was reduced by 40%.

### Statistical analysis

First, patients were divided into two groups: (i) patients who developed AKI and (ii) patients without AKI. The distribution was calculated with the Kolmogorov Smirnov test. Quantitative variables were analysed with the Student's *t* test and presented with their mean and standard deviation or median and interquartile range. Qualitative variables were analysed with Chi-squared test or Fisher's exact coefficient, and presented with their frequency distribution. Bivariate and multivariate analyses were obtained with logistic regression to identify risk factors for AKI after CAR T-cell therapy. As a second step, actuarial survival curves were estimated using the Kaplan–Meier method to identify risk factors for mortality in a 365-day follow-up. Statistical studies were analysed using the SPSS version 20 program. Odds ratio (OR) for AKI with 95% confidence interval (CI) was reported. Two-sided *P* values 0.05 were considered statistically significant.

## RESULTS

### Patient characteristics 

Median age was 61 years [range, 20–81] and 66% were male. Demographic and clinical characteristics for all patients are shown in Table 1. Briefly, hypertension was present in 37% of patients, diabetes in 8%, and cardiovascular disease in 5%. CKD was present in 11 (9%) patients due to obstructive nephropathy related to a retroperitoneal mass (5), unknown kidney disease (5) or chemotherapy-related toxicity (1). Hematological neoplasms included diffuse large B-cell lymphoma (91%), B-cell acute lymphoblastic leukemia (5%), mantle cell lymphoma (4%), and primary mediastinal large B-cell lymphoma (1%). The median of previous treatment lines was 2 [IQR, 1–3], 29 (25%) patients had undergone an autologous hematopoietic cell transplant (HCT), and 1 (1%) patient an allogeneic HCT. Fourteen (12%) patients required fludarabine dose adjustment for a decreased glomerular filtrate (<60 ml/min/1.73 m^2^ in 11 patients and <70 ml/min/1.73 m^2^ by Cockcroft–Gault formula in three patients). Regarding the type of CAR-T construct, 50% of patients received tisagenlecleucel, 28% lisocabtagene maraleucel, 20% axicabtagene ciloleucel, and 2% brexucabtagene autoleucel.

**Table 1:** Demographic characteristics of the full patient population.

Variables	All	AKI patients (*n*=24)	Non-AKI patients (*n*=91)	*P* value
Age, median [range]	61 [20-81]	16 (66.7)	46 (50.5)	0.16
Males, *n* (%)	76 (66)	20 (83.3)	56 (61.5)	**0.04**
Hypertension, *n* (%)	43 (37.4)	13 (54.2)	30 (33.0)	0.05
Type 2 diabetes mellitus, *n* (%)	9 (7.8)	3 (12.5)	6 (6.6)	0.34
Chronic kidney disease, *n* (%)	11 (9.5)	6 (25.0)	5 (5.5)	**0.004**
Dose reduced fludarabine, *n* (%)	14 (12.2)	8 (33.3)	6 (6.6)	**0.002**
CAR T-cell construct, *n* (%)				**0.04**
Lisocabtagene maraleucel	32 (27.8)	7 (29.2)	25 (27.5)	
Tisagenlecleucel	57 (49.6)	14 (58.3)	43 (47.3)	
Axicabtagene ciloleucel	23 (20.0)	1 (4.2)	22 (24.2)	
Brexucabtagene autoleucel	3 (2.6)	2 (8.3)	1 (1.1)	

### AKI after CAR-T cell therapy

A total of 24 (21%) patients developed AKI after CAR T-cell therapy. Onset of decreased renal clearance took place during LD chemotherapy (with fludarabine and cyclophosphamide) in two patients and after CAR T-cell infusion in 22 patients. The latter group included three patients at day +1, 13 patients at day +7, one patient at day +14, two at day +21, and three patients at day +28. Among these cases, 17 (14.8%) had AKI stage 1, four (3.5%) had AKI stage 2, and three (2.6%) had AKI stage 3 (Table S1 and Table S2, see online supplementary material). Two patients required renal replacement therapy with a peak serum creatinine at day +7 of 2.45 and 2.0 mg/dL, respectively. One of whom died within one month from AKI stage 3 onset due to CRS grade 4 and refractory septic shock, and the last patient recovered kidney function within the first month after CAR-T cell therapy, achieving a serum creatinine of 0.4 mg/dL.

In terms of the AKI cohort, 19/24 (79%) patients recovered their baseline kidney function within the first month after CAR T-cell infusion [median 1 week, range 1–4 weeks]. The remaining five patients continued to present an impaired kidney function after two months of follow-up (4/5 diagnosed with new AKI onset) [median creatinine 1.4 mg/dL, range 1.3–2.4 mg/dL]. Regarding the CKD group, five out of the six patients who developed AKI recovered baseline kidney function within 30 days of CAR T-cell infusion, while the remaining patient developed a decreased creatinine clearance secondary to CRS grade 2 (from 34 ml/min/1.73 m^2^ to 27 ml/min1.73 m^2^) and maintained it after the 30-day of follow-up period. There were no significant differences in ICU admission and/or mortality in this CKD group.

### Changes in serum electrolytes

In our cohort there were no cases of severe electrolyte disorders. The rate of hyponatremia (sodium <135 mmol/L) reached 15%, hypophosphatemia (phosphate <2.5 mg/dL) in 22% and hypocalcemia (calcium <8.5 mg/dL) in 43% by day +7, hypokalemia (potassium <3.5 mmol/L) in 21% and hypomagnesemia (magnesium <2 mg/dL) in 74% by day +21. In comparison between the AKI and non-AKI groups, hypophosphatemia and hypocalcemia by day +1 (*P* = 0.002 and *P* = 0.01, respectively), hyponatremia and hypocalcemia by day + 7 (*P* = 0.001 and *P* = 0.03, respectively), hypocalcemia by day +14 (*P* = 0.03), and hyponatremia by day +21 (*P* = 0.04) showed significant differences in the univariable analysis. However, these differences were not seen in the adjusted multivariable analysis.

### CAR T-cell therapy complications and mortality

The most frequent CAR T-cell related adverse events were CRS (82% any grade and 30% CRS grade ≥2) and ICANS (29% any grade, 4% grade ≥3) (see Table 2). To manage these adverse events, 37 (32%) patients required tocilizumab and 34 (30%) patients received steroids. Six patients were admitted to the ICU (one with the diagnosis of refractory septic shock, four for CRS grade ≥2 associated to ICANS grade ≥2, and one for CRS grade ≥3). Median ICU stay was 8 days [range, 2–30 days]. Thirty-six patients died during study follow-up, 31 due to disease progression and five related to CAR-T cell therapy, including four cases of bacterial refractory septic shock and one case of heart failure.

**Table 2:** Clinical outcomes after CAR T-cell infusion in the 115 patients.

Variables	All	AKI patients (*n*=24)	Non-AKI patients (*n*=91)	*P* value
ICANS any grade, *n* (%)	33 (28.7)	11 (45.8)	22 (24.2)	**0.04**
ICANS grade ≥3, *n* (%)	4 (3.5)	3 (12.5)	1 (1.1)	**0.03**
CRS any grade, n (%)	95 (81.9)	21 (87.5)	74 (81.3)	0.56
CRS grade ≥2, *n* (%)	35 (30.2)	15 (71.4)	20 (27)	**0.001**
Steroid therapy, *n* (%)	34 (29.6)	19 (62.5)	15 (20.9)	**0.001**
Tocilizumab, *n* (%)	37 (32.2)	16 (66.7)	21 (23.1)	**0.001**
ICU admission, *n* (%)	6 (5.2)	3 (12.5)	3 (3.3)	0.07
Death, *n* (%)				**0.005**
Due to disease progression	31 (86.1)	3 (50.0)	28 (93.3)	
Non relapse related mortality	5 (13.9)	3 (50)	2 (6.7)	

CRS: cytokine release syndrome; ICANS: immune effector cell-associated neurotoxicity syndrome; ICU: intensive care unit.

### Risk factors for AKI and death

In the bivariate analysis, male gender (*P* = 0.04), previous history of CKD (*P* = 0.004), ICANS grade ≥3 (*P* = 0.03), CRS grade ≥2 (*P* = 0.001), and tocilizumab treatment (*P* = 0.001) were associated with AKI. In the multivariable analysis, male gender [adjusted odds ratio (aOR) 6.54, 95% CI 1.14–37.6, *P* = 0.04], CKD [aOR 8.20, 95% CI 1.52–44.3, *P* = 0.02], ICANS grade ≥3 [aOR 29.9, 95% CI 1.50-596.9, *P* = 0.03], and CRS grade ≥2 [aOR 3.98, 95% CI 1.18–13.4, *P* = 0.03] remained significant risk factors for AKI (Table 3). In the actuarial survival analysis one year after CAR T-cell infusion for 98/115 patients, CRS grade ≥2, ICANS any grade, hemoglobin levels and creatinine at day +1, +7, +28 showed no statistically significant impact on mortality (Fig. S2, see online supplementary material).

**Table 3:** Adjusted odds ratio for AKI development.

Variable	aOR	CI 95%	*P* value
Age ≥61 years	2.24	0.66–7.65	0.20
Males	6.54	1.14–37.6	**0.04**
CKD	8.20	1.52–44.3	**0.02**
ICANS grade≥3	29.9	1.50–596.9	**0.03**
CRS grade≥2	3.98	1.18–13.4	**0.03**

aOR: adjusted odds ratio; CKD: chronic kidney disease; CRS: cytokine release syndrome; ICANS: immune effector cell-associated neurotoxicity syndrome.

Multivariate analysis was obtained with logistic regression.

### Outcomes of patients with new AKI onset development and previous history of CKD

At a median follow-up of 296 days, the mean progression-free survival (PFS) was 198 days [95% CI, 100 to 297 days] in AKI patients vs 161 days [95% CI, 115 to 208 days] in non-AKI patients (*P* = 0.72). The mean overall survival (OS) was 281 days [95% CI, 27 to 227 days] in AKI patients versus 300 days [95% CI, 278 to 323 days] in non-AKI patients (*P* = 0.77).

**Figure 2:**
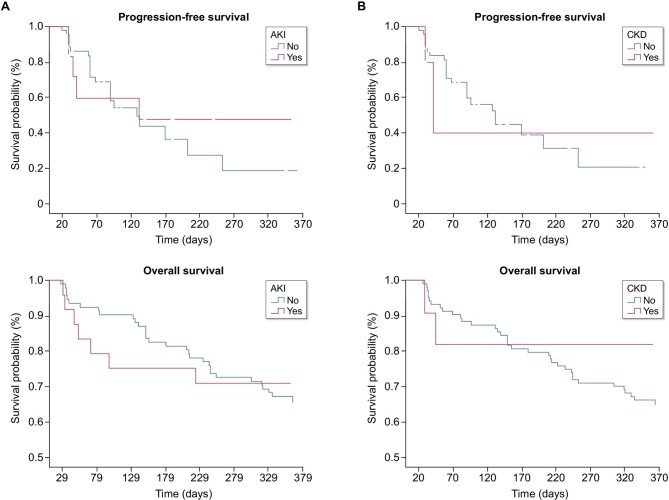
Clinical outcomes in patients with AKI and previous history of CKD. (**A**) PFS and OS stratified by AKI development. (**B**) PFS and OS stratified by CKD.

The median PFS was 41 days [95% CI, 25 to 57 days] in CKD patients vs 132 days [95% CI, 81 to 182 days] in normal kidney function patients (*P* = 0.94). The OS was 305 days [95% CI, 230 to 380 days] in CKD patients versus 295 days [95% CI, 273 to 317 days] in normal kidney function patients (*P* = 0.33) (Fig. 2).

## DISCUSSION

CAR T-cell therapy has been a revolutionary treatment for R/R hematologic malignancies but it can lead to well-known severe complications. In our study, 24 (21%) patients developed AKI after CAR T-cell therapy, generally in the first week after infusion. Even though AKI is a complication of which treating physicians must be aware, it presents mostly with a mild disease and a rapid recovery, demonstrating that AKI is usually transient after CAR T-cell therapy. Our results are similar to those obtained by Gutgarts *et al.*, who described AKI in 14 (30%) out of 46 patients with non-Hodgkin lymphoma receiving CAR T-cell therapy in the first 100 days after infusion. The most frequent presentation of AKI in this cohort was grade 1. In addition, 3 (21%) of the 14 AKI-patients died and 10 (71%) recovered baseline kidney function in a 30-day period [34]. Another study by Gupta *et al.* described 15/67 (22%) patients diagnosed with diffuse large B-cell lymphoma who developed AKI within 30 days of CAR T-cell infusion. The authors confirmed that AKI was usually a mild disease with fast recovery [35]. The largest study to date, led by Hanna *et al.*, compared the risk of AKI in 232 patients who received CAR T-cell therapy with 414 patients who underwent auto-HCT, confirming no significant differences between both groups in the first 30 days after infusion [38]. These results are similar to our own findings, indicating that AKI associated with CAR T-cell infusion is a mild complication with a fast recovery.

Despite the lack of consensus on the optimal fludarabine dose reduction in patients with renal impairment, the incidence of AKI related to fludarabine exposure remains lower than 5% [44]. The need for dose adjustment in CKD patients relies on the 60% of drug excreted through urine [45]. Most studies suggest a 20–25% reduction for mild kidney impairment and up to a 50% reduction for moderate to severe impairment [46–48]. Wood *et al.* evaluated the outcomes of CAR T-cell therapy in patients with renal impairment and determined that progression-free survival and overall survival did not differ between patients with and without renal impairment or between those who received standard-dose fludarabine and those who received reduced-dose [49, 50]. Wood *et al.* also described that baseline renal function did not affect renal or efficacy outcomes after CAR T cell therapy. In contrast, in our study we found that patients with previous CKD had higher risk of AKI development, similarly to those described by Lyu *et al.* [50]. Our analysis also suggests that there were no differences in AKI development between standard and dose-reduced fludarabine after the multivariable analysis in accordance with Wood *et al.* [48].

Our study is the first that identifies male gender as an independent risk factor for AKI development. Furthermore, the most frequent complications after CAR T-cell infusion were CRS and ICANS. AKI was diagnosed in 88% of patients with CRS, and 46% in patients with ICANS. CRS grade ≥2 and ICANS grade ≥3 were adverse events identified as risk factors for AKI development. Similarly, other authors established CRS grade ≥3 as an independent risk factor for AKI [34, 35]. On the other hand, Ahmed *et al.* carried out a retrospective analysis of the impact of CKD and AKI on CAR-T outcomes in adult patients with non-Hodgkin’s lymphoma, observing that CKD patients had an increased frequency of CRS and ICANS [52]. Furthermore, Ahmed *et al.* defined ICANS ≥2 was an independent risk factor for AKI development [52]. We can surmise that proinflammatory status after CAR T-cell therapy may decrease renal perfusion and subsequently favor prerenal AKI development. Despite the risk of AKI with the proinflammatory state after CAR T-cell therapy, we consider that the potential benefit of the therapy warrants that clinicians actively monitor the development of CRS and/or ICANS.

Electrolyte disorders such as hypophosphatemia, hyponatremia, and hypokalemia are common after CAR T-cell therapy [34]. Farooqui *et al.* reported in their study 14/83 (17%) patients developing AKI within one month after CAR-T infusion. Moreover, they found that both absolute and relative from baseline to peak levels in lactate dehydrogenase were higher among AKI patients compared to non-AKI patients [53]. We observed that lower sodium, calcium, and phosphorus blood levels were associated with AKI in the univariable analysis. However, these findings were not confirmed in the multivariate analysis. This may be in part related to the lack of some laboratory assessments during the study follow-up.

Our study has some limitations, besides its retrospective design which precludes inferences of causal associations and selection bias. All patients included in the study received the CAR T-cell infusion, potentially overestimating the survival benefit of this treatment (immortal bias). Fludarabine dose adjustment was established according the Cockcroft–Gault formula even though we defined CKD based on the KDIGO definition (based on CKD-EPI formula). Blood test results could not be systematically compared as not all data were collected in our patient population. However, one of the strongest points of this study, in comparison with previously published articles, is that we had one of the largest cohorts and carried out long-term follow-up. Further multicenter studies with extended follow-up are needed to confirm our findings.

## CONCLUSION

In our study, 21% of patients developed AKI, usually in the first week after CAR-T infusion. Most of the patients (79%) recovered kidney function within the first month post-treatment, suggesting that AKI is frequent but mild, with a fast recovery in this setting. Male gender and a history of CKD, together with development of CRS grade ≥2 and/or ICANS grade ≥3 after CAR T-cell infusion were identified as independent risk factors for AKI development.

## Supplementary Material

sfae027_Supplemental_File

## Data Availability

The data underlying this article are available in the article itself.
