# Supplementary material for: Transient acute kidney injury after chimeric antigen receptor T-cell therapy in patients with hematological malignancies
Source: Clin Kidney J. 2024 Feb 20;17(3):sfae027. doi: 10.1093/ckj/sfae027 (PMC10946657; doi:10.1093/ckj/sfae027)

Supplemental data

Table S1. Acute kidney injury score and mortality

Variables	All	Alive	Deceased	p value
Non-AKI patients, n (%)	91 (79.1)	61 (77.2)	30 (83.3)	0.16
AKI patients (Akin score)				
- 1, n (%)	17 (14.8)	13 (16.5)	4 (11.1)	
- 2, n (%)	4 (3.5)	4 (5.1)	0 (0)	
- 3, n (%)	3 (2.6)	1 (1.3)	2 (5.6)	

Table S2. Mean serum creatinine values during the 30 days follow-up in patients treated with CAR-T cells therapy.

**Serum Creatinine (mg/dL)**	**AKI patients**	**Non-AKI patients**
**Day +1**	1.14 ± 0.38	0.70 ± 0.23
**Day +7**	1.52 ± 0.70	0.75 ± 0.25
**Day +14**	1.22 ± 0.56	0.74 ± 0.27
**Day +21**	1.19 ± 0.58	0.77 ± 0.26
**Day +28**	1.12 ± 0.53	0.76 ± 0.25

Figure S1. Etiology of chronic kidney disease in our 11/115 patients.

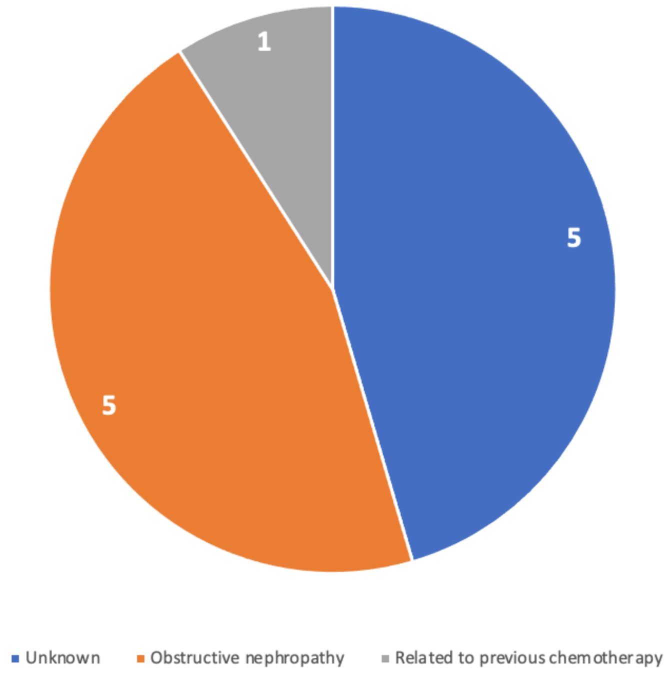

Figure S2. One-year follow-up time for the relationship of creatinine and mortality analyzed with Kaplan Meier curves (Log-rank) based on analytical results of the 98/115 patients treated with CAR-T cell therapy.

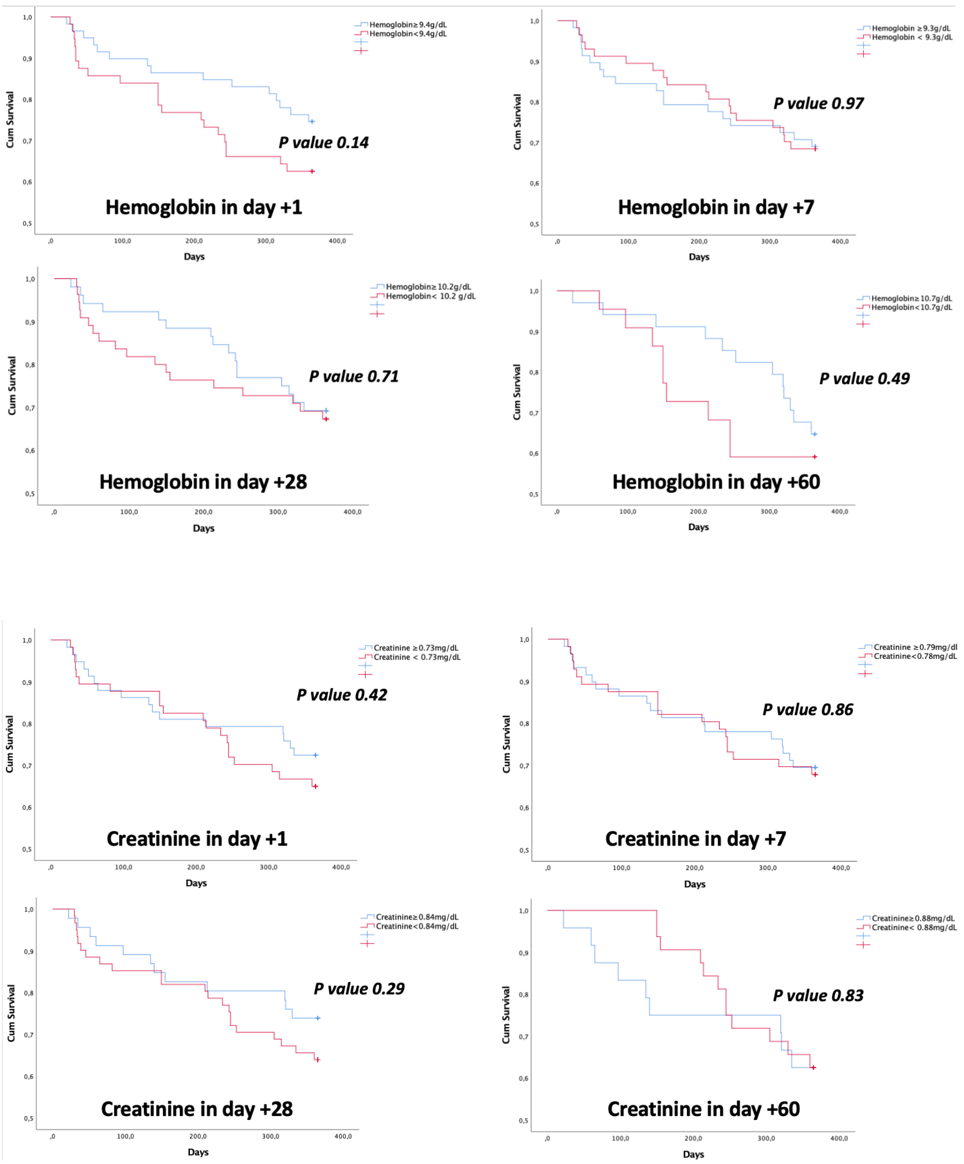

Supplement: sfae027_Supplemental_File [file sfae027_supplemental_file.pdf]
